# Advancing Ocular Medication Delivery with Nano-Engineered Solutions: A Comprehensive Review of Innovations, Obstacles, and Clinical Impact

**DOI:** 10.7759/cureus.66476

**Published:** 2024-08-08

**Authors:** Praiseth Bellston Rajan, Jebastin Koilpillai, Damodharan Narayanasamy

**Affiliations:** 1 Pharmacy, SRM Institute of Science and Technology, Chennai, IND; 2 Pharmaceutics, SRM Institute of Science and Technology, Chennai, IND

**Keywords:** toxicity, safety, drug penetration, ocular drug delivery, nanotechnology, nanocarriers

## Abstract

Recent advancements in ocular drug delivery have led to the introduction of a range of nanotechnology-based systems, such as polymeric nanoparticles, solid lipid nanoparticles, nanostructured lipid carriers, inorganic nanoparticles, niosomes, liposomes, nanosuspensions, dendrimers, nanoemulsions, and microemulsions. These systems enhance drug retention, penetration, bioavailability, and targeted delivery, promising prolonged drug release, and improved patient compliance. However, their interactions with biological systems pose potential toxicity risks, necessitating a careful evaluation of nanoparticle size, shape, surface charge, and coating. Traditional ocular drug delivery methods, like topical applications and injections, face challenges due to anatomical and physiological barriers, leading to frequent dosing and systemic toxicity risks. Nanocarriers offer solutions by improving drug permeation and targeted delivery, yet translating these innovations from research to clinical practice involves overcoming hurdles related to manufacturing scale-up, quality control, regulatory approval, and cost-effectiveness. The quality by design (QbD) framework provides a systematic approach to optimize nanocarrier formulation and process design, ensuring safety and efficacy. Assessing the safety of nanocarriers through in vivo and in vitro studies is crucial for their clinical application. This review explores the use of various nanomedicines in ocular drug delivery, highlighting the current state of ocular medication delivery and considering critical aspects such as scaling up and clinical applications.

## Introduction and background

In recent years, a variety of delivery techniques have been created to tackle the major challenges associated with ocular drug delivery, aiming to develop a safe and effective system for administering medication to the precise site. The emergence of various nanotechnology-based nanomedicines and novel systems, including polymeric nanoparticles (NPs), solid lipid nanoparticles (SLN), nanostructured lipid carriers (NLC), inorganic nanoparticles, niosomes, liposomes, nanosuspensions, dendrimers, nanoemulsions, and microemulsions, has resulted in longer retention periods, better drug penetration through ocular barriers, improved bioavailability, and targeted delivery of drugs to particular cells and tissues [1]. The constraints of conventional and sustained-release ocular dosage forms have been addressed in the last several decades by the widespread application of nanotechnological interventions to improve drug release, bioavailability, and penetration of ocular drugs.

Ocular delivery systems based on nanotechnology serve as drug depots that provide prolonged drug release, lower the frequency of administration, and improve patient compliance. Additionally, the encapsulated active compounds are shielded from enzymatic and metabolic breakdown by nanocarriers. The negative aspects of conventional ocular dosage forms, such as ophthalmic drops, ointments, injections, and implants, are addressed by the developed nanotherapies. Because of physiological and anatomical barriers, these conventional forms have low penetration, reduced bioavailability, drug breakdown by enzymes, and general protein binding, all of which are efficiently addressed by nanotherapies [2,3]. When used as ophthalmic drops, submicron-sized liposomes have recently shown promise as a topical medication delivery technique for treating conditions affecting the posterior portion of the eye [4].

Nanotoxicology focuses on investigating the safety and toxicity of nanoparticles, including their interactions with biological systems. Due to their small form factor and significant surface area, these nanomaterials interact rapidly and effectively with cells and subcellular structures, potentially leading to harmful effects. Nanoparticles (NPs) often get trapped in the retinal area, vitreous cavity, and on the ocular surface upon administration, posing potential harm to the eyes. Because the eye is a surface organ, it is particularly susceptible to environmental nanoparticle toxicity. Furthermore, the toxicity of nanoparticles is closely linked to factors such as their size, shape, surface charge, and coating. For example, liver cells showed greater toxicity to 15 nm gold nanoparticles compared to 60 nm gold nanoparticles [5]. Therefore, it is crucial to investigate the potential ocular toxicity of nanoparticles used in ophthalmology. Nanotechnology-based ocular delivery systems (NODS) serve as drug reservoirs, which improve patient compliance, lower the frequency of administration, and provide prolonged drug delivery. In addition, the encapsulated active substances are shielded from enzymatic and metabolic breakdown by nanocarriers. Most research on the safety of liposomes focuses on pharmaceutical aspects such as stability, zeta potential, particle size, and properties related to their release in vivo. There are fewer reported systemic complaints regarding irritation of ocular tissues, suggesting that liposomes may not cause stimulating effects on the eyes. This hypothesis is supported by the use of certain liposome formulations in treating dry eye conditions [6,7]. 

Nano-drug delivery systems are considered the most effective method for targeted drug delivery [2-4]. Nevertheless, ensuring the safety and maintaining quality control of these nanocarrier drug delivery systems have become increasingly challenging. Challenges faced include the physicochemical characteristics, lack of reproducibility, structural destabilization, and high production costs of nano-drug delivery systems. Addressing these effectively requires optimizing nano-formulation and process design using scientific methodologies. Quality by design (QbD) provides a systematic approach with predefined objectives, employing scientific and risk-based proactive methods [8,9].

The emergence of nanotechnology has brought about substantial progress in ocular medication delivery, introducing innovative therapeutic strategies for treating eye conditions. It is expected that nanotechnology-based ocular drug delivery will eventually become the predominant approach in ocular therapy despite most current research being in the early stages [1-3,6].

## Review

Conventional ocular drug administration routes

Drugs can be administered to the eye using various methods, including topical preparations like eye drops and ointments, as well as through intravitreal, subconjunctival, and periocular injections, and systemic routes such as oral and parenteral administration. 

Topical Administration

Conventional dosage forms such as ophthalmic drops, suspensions, and ointments are frequently applied topically due to their convenience for patients. Generally, these medications are absorbed through the corneal and conjunctival routes. However, factors like tear dynamics, loss before reaching the cornea, and tearing result in only a small fraction (approximately 5%) of the drug reaching the surface of the eye. Additionally, anatomical barriers further limit drug absorption, contributing to the drug's low bioavailability. As a result, frequent application of eye drops is required to maintain sufficient drug levels on the ocular surface. Ointments, on the other hand, may reduce patient compliance by limiting the duration of light exposure to the ocular surface Moreover, excessive tear production during inflammation dilutes the drug and rapidly washes it away from the affected area [1,3,4].

Systemic Administration

Drugs can be administered systemically to the eyes through parenteral and oral routes. Only 1% to 2% of the medication reaches the retinal and vitreous portions of the eye due to the retina's strong barriers and the eye's relatively small blood flow compared to the rest of the body. Therefore, in order to obtain therapeutic effects, frequent dosing is necessary, and higher doses raise the risk of systemic toxicity. Children and infants are especially susceptible to systemic side effects because they have lower levels of P-glycoproteins in their blood-brain barrier. As a result, there are many difficulties with administering medication systemically to the posterior portion of the eye [1,4].

Ocular Injections

**Figure 1:**
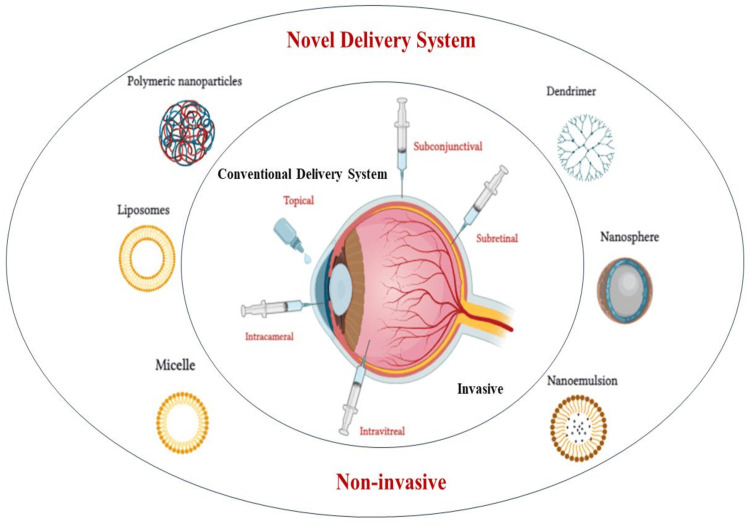
Novel ocular drug delivery systems This image was created by Dr. Praiseth Bellston Rajan.

Intravitreal injection with a 27 or 30 gauge needle is the main technique for injecting 20-100 µl of either a suspension or solution into the vitreous cavity. Drug distribution within the gel-like substance that makes up the vitreous cavity depends on a number of factors, including the molecular mass of the drug and the pathophysiological circumstances of the vitreous area. Larger linear molecules (greater than 40 kDa) and globular molecules (greater than 70 kDa) are kept in the vitreous cavity for longer periods of time than smaller drug molecules (with a molecular mass less than 500 Da). Hyaluronan, a negatively charged glycosaminoglycan found in the vitreous, can interact with molecules that are positively charged. A needle is inserted into the eye during an intravitreal injection, which can result in major risk and complications such retinal detachment, cataracts, infections, and vitreous hemorrhage. In comparison to intravitreal injections, intracameral, subconjunctival, and posterior juxtascleral injections are thought to be less invasive. However, these methods have drawbacks such as lower patient compliance, increased costs, and being perceived as both painful and invasive [1,3,4]. The various modern and classic ocular methods of drug administrations are shown in Figure 1.

Translational hurdles in ophthalmic nanomedicine

The transition of ophthalmic nanomedicine from research to clinical use is fraught with several complex challenges. The main issues pertain to the difficulties in effectively delivering drugs to the different tissues within the eye. Significant obstacles include physiological barriers like blinking and tear turnover, anatomical barriers such as the multiple layers of the cornea and sclera, and dynamic barriers like tear drainage and conjunctival blood flow, all of which hinder optimal drug delivery [3,10]. Furthermore, the presence of tight junctions in the blood-retinal barrier restricts the access of systemically administered drugs to the retina, making effective treatment delivery even more challenging.

Nanocarriers have emerged as a promising solution to overcome these challenges by improving drug permeation and facilitating targeted delivery to specific ocular sites. Nevertheless, the transition of nanomedicine into clinical practice faces additional obstacles. These hurdles encompass scaling up production processes, ensuring stringent quality control standards, navigating intricate clinical development pathways, addressing biocompatibility and safety concerns, meeting rigorous regulatory requirements, and establishing cost-effectiveness relative to current therapies [1]. Effectively overcoming these multifaceted challenges is essential to advance ophthalmic nanomedicine from theoretical research to practical clinical solutions for disorders related to the eye.

Delivering drugs to the eye is exceedingly challenging due to the intricate and varied physiological barriers. Consequently, several innovative delivery mechanisms have been developed to address this issue. A small number of these novel drug delivery techniques like liposomal drug delivery system, nanomicelles, polymeric nanoparticles, nanocrystals, nanoemulsions, hydrogel-based nanoparticles are some of the successful techniques for treating different eye disorders that have been currently successfully commercialized as nano-formulation delivery systems despite intensive research. The marketed product must meet acceptable standards in terms of patient acceptability, safety, efficacy, and stability [1,3,10].

Additionally, research has shown that clinical translation of many of these drugs is hampered when nano-based delivery systems are scaled up from laboratory to huge industrial scales. For example, increasing the size of nanotechnology-based delivery systems like nanoparticles prepared using less energy-intensive methods like phase inversion temperature, phase inversion composition, and emulsion inversion point methods often results in modifications of their many attributes, most notably their physicochemical properties. In response to the limitations of low-energy processes, several high-energy techniques were developed. However, it has been noted that the production of nanoparticles by powerful techniques such as heat-assisted homogenization and ultrasonic treatment might lead to coalescence, which can cause thermodynamic instability in the system [1,7,10].

Furthermore, investigations reveal that numerous approaches for developing these nano-based technologies typically entail complex, step-by-step processes. These techniques also have very poor reproducibility and inadequate consistency. Developing nano-based technologies is very difficult due to issues like batch-to-batch variance and dispersion strength, which complicate quality control [3].

It is critical to increase the stability as well as safety of nanocarriers in order to mitigate any problems. Many new drug delivery methods have not undergone extensive in vivo testing in human eyes, having been primarily evaluated in vitro or on animals. The metabolic fate of nanocarriers in the eye remains unclear, and their targeting capabilities need enhancement. Due to their low cost and ease of handling, rodents have been widely utilized in preclinical research. In particular, these studies have made considerable use of rats, mice, and rabbits. Nevertheless, it has been observed that the pharmacokinetic characteristics of different formulations can vary significantly due to variations in ocular anatomy and physiology. For instance, rodents typically have higher lens-to-cornea ratios and smaller eyes compared to humans. Moreover, rabbits produce more mucus, blink less frequently, and are prone to ocular irritation. Additionally, the immunological composition of the human retina differs significantly from that of the rodents commonly used in preclinical studies. Due to these factors, predicting the success of clinical studies based on animal-based preclinical testing is exceedingly challenging [1,3,10].

QbD approaches for the nano-formulations 

The development of nanocarriers necessitated the inclusion of multiple components, all essential for ensuring the formulation's efficacy and safety. The development process involves both "bottom-up" and "top-down" approaches, each comprising several stages. Factors such as surfactant levels, volume of the surfactant solution, duration and intensity of mixing, exposure time for size reduction, and temperature conditions are among the variables that can influence either method. Understanding how each ingredient interacts with the others as well as how process variables affect even small variations in component concentrations is essential [8,9]. Achieving large-scale manufacture of nanocarrier formulations with the specified quality demands a thorough understanding of how slight changes in each element and process factors interact. 

**Figure 2:**
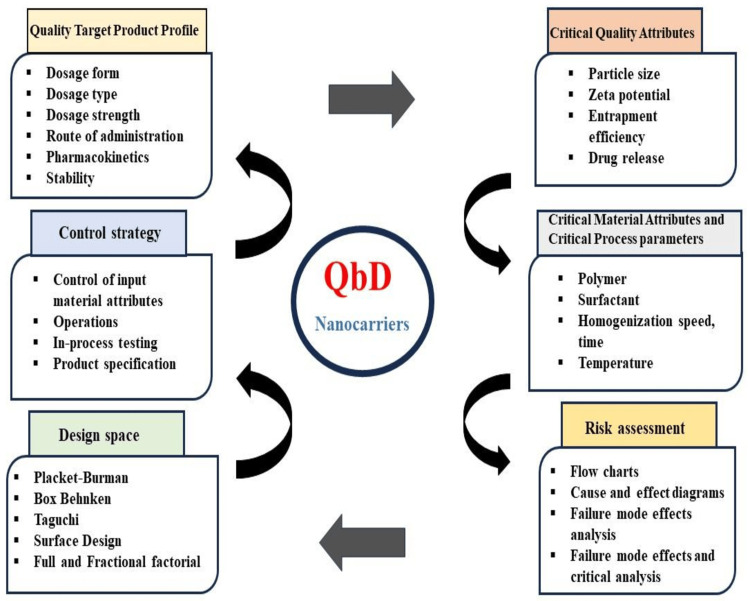
Components of quality by design This image was created by Dr. Praiseth Bellston Rajan.

Thus, employing the quality by design (QbD) framework for developing nanomaterials plays a crucial role in identifying key manufacturing parameters and controlling factors that influence the safety and quality of the final product. In drug delivery, QbD aims to determine significant product quality standards from safety, effectiveness, and clinical performance. Ultimately, it enhances process capability by minimizing product variability through the systematic evaluation of variable elements in materials and processes. Risk evaluation along with control are prioritized in QbD to guarantee the applicability of products and processes [8,9]. The elements of QbD are shown in Figure 2.

Quality Target Product Profile

The Quality Target Product Profile (QTPP) is a predetermined list of requirements for a drug that guarantees both the safety and effectiveness of the product as intended. It also assists in determining the essential qualitative aspects of the product. Before developing the QTPP, a connection must be established between the product's physicochemical properties and its biological performance as outlined in the target product profile. A nanoformulation's distribution of nanocarriers to the intended target cell, tissue, or organ, the formulation's pharmacokinetics, the toxicological characteristics of the drug product, and the nanocarriers' safety and efficacy profile are usually important components of its biological performance [8]. The quality target product profile for nano-formulations is listed in Table 1.

**Table 1:** Quality target product profile for nano-formulations QTTP, Quality target product profile.

QTPP	Objective	Reasoning	Reference
Drug formulation	Nanotechnology formulation	To improve the drug’s bioavailability, penetration, stability, and efficacy, which are dependent on the drug and dosage	[8]
Method of delivery	Topical, injectable, or oral	Targeted, non-invasive, simple to administer, and with less adverse effects	[8]
Physical appearance	Lyophilized gel or powder	Easy administration ensured by the final product's physical appearance	[8]
Physicochemical evaluation	Entrapment efficiency, particle size, zeta potential, drug release	To ensure the drug is effectively loaded into the nanosystem. Affects penetration and absorption, impacts the stability of the product, and affects the drug's pharmacokinetic properties	[8]
Active pharmaceutical ingredient’s solubility in the drug delivery system	Elevated to 50%	Influences the drug release schedule, therapeutic outcomes, and rate of absorption into the skin	[8]
Viscosity of dispersion	100–500 mPas	Impact on the stability and release of drugs	[8]
pH	Compatible with the formulation	Effect on drug loading, release, penetration rate, and stability	[8]
System for closing containers	Suitable for the dosage form	To ensure desired shelf lives	[8]
Stability	Quality criteria	Effect on product quality	[8]
Pharmacokinetics	Absorption, distribution, metabolism, and targeting	Essential to attain the intended effect	[8]

Critical Quality Attributes

**Figure 3:**
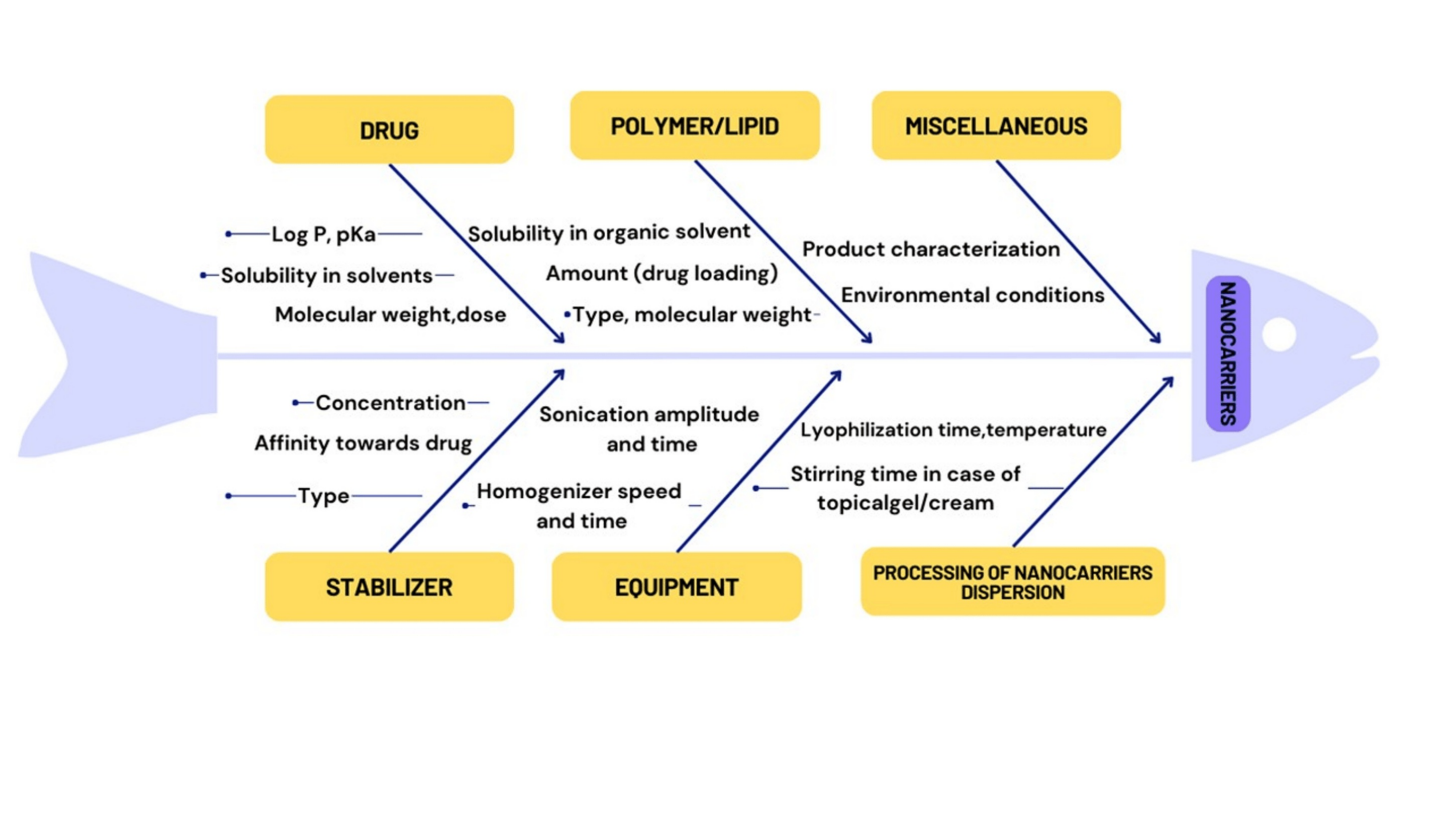
An Ishikawa diagram, or fishbone diagram, shows possible formulation and procedure variables influencing the CQAs of a nano-formulation This image was created by Dr. Praiseth Bellston Rajan.

Critical Quality Attributes (CQAs) direct the creation of procedures as well as products and are developed from the Quality Target Product Profile (QTPP). They establish the acceptable range or limits for quality, ensuring that the nanoproduct meets the intended high standards. CQAs link the therapeutic performance of a product to its physical, chemical, biological, and microbiological properties. These include drug release, stability, impurity profile, entrapment efficiency, assay, sterility, and remaining solvents if organic solvents were employed in the manufacture of nanocarriers [8,9]. A fishbone diagram, also called an Ishikawa or cause-and-effect diagram, is useful for identifying all possible formulation and process parameters that affect the CQAs of a nano-formulation (Figure 3).

Critical Quality Attributes for nanocarriers are listed in Table 2.

**Table 2:** Critical Quality Attributes for nanocarriers CQA, Critical Quality Attributes

CQAs	Objective	Reasoning	Reference
Size of the particle	<250nm	A smaller globule size facilitates simple passage through the cellular membrane and aids in cancer cell targeting and passive targeting for brain administration.	[8]
Zeta potential	> ±30mV	The ideal zeta potential value is believed to be crucial since it is necessary for both particle homogeneity and dispersion stability.	[8]
Encapsulation efficiency	Maximum elevation	Administering the intended dosage, accomplishing regulated drug release from nanocarriers, and optimizing therapeutic response all depend on better entrapment efficiency. The necessary volume for administration may also be lowered by increased drug loading.	[8]
Drug release	Prolong release (>12h)	For the objective of achieving prolonged medication absorption, slow and predetermined release is needed and is therefore deemed crucial.	[8]

Critical Material Attributes

The key elements influencing the variation of Critical Quality Attributes are Critical Material Attributes, which are closely linked to the composition of nanocarrier formulations. These factors include physical and chemical, biopharmaceutical, and microbiological characteristics of the input materials, which must stay within predetermined bounds in order to guarantee the desired purity of the final product. The important Critical Material Attributes (CMAs) in nanocarriers include variables such as the type, volume, and concentration of surfactants; the concentration of organic solvents (in solvent evaporation techniques); the levels of polymers and lipids; and the type and amount of salts used to prepare buffers or modify particle charge [8]. Critical Material Attributes and risk assessment for nanocarriers are listed in Table 3.

**Table 3:** Critical Material Attributes and risk assessment for nanocarriers

	Critical Material Attributes				
Critical Quality Attributes of a drug product	Drug	Solid lipid/ liquid lipid	Polymer	Surfactant/stabilizer concentration	Reference
Size and Polydispersity Index	Decreased	Moderate	Moderate	Increased	[8]
Encapsulation efficiency	Increased	Increased	Increased	Moderate	[8]
Release of the drug	Moderate	Increased	Increased	Decreased	[8]
Zeta potential	Decreased	Decreased	Moderate	Increased	[8]

The rationale for the initial risk evaluation of material properties is listed in Table 4.

**Table 4:** Rationale for the initial risk evaluation of material properties CMA, Critical Material Attributes

Critical Quality Attributes of a drug product	CMA	Rationale
Size and Polydispersity Index	Drug	Size is not significantly affected by drug characteristics when the drugs are dissolved or distributed in lipid or polymeric solutions.
	Solid lipid	Small changes in size and Polydispersity Index can result from fluctuations in lipid content. If more lipid is present, particle size could grow if it is not fully used.
	Liquid lipid	The size of nanocarriers is not significantly impacted by liquid lipid.
	Concentration of the surfactant	The surfactant has two crucial roles in reducing the size: provide stability to nanocarriers and inhibit particle agglomeration.
Encapsulation efficiency	Drug	Drug characteristics have an impact on encapsulation because drugs that are very lipophilic are more likely to encapsulate.
	Solid lipid	The right amount of lipid is needed for better effectiveness in encapsulation.
	Liquid lipid	Liquid lipid enhances the efficacy of drug entrapment.
	Concentration of the surfactant	Drug entrapment in surfactants is low.
Release of the drug	Drug	The properties of the drugs have a modest effect on the release of drugs.
	Solid lipid	Lipid encapsulation of the drugs allows for regulated drug release.
	Liquid lipid	The impact of liquid lipid content on entrapment efficiency is moderate.
	Concentration of the surfactant	Drug release is not significantly influenced by the surfactant.
Zeta potential	Drug	Zeta potential is not significantly affected by the drug.
	Solid lipid	Zeta potential is partially influenced by lipid charge.
	Liquid lipid	There is minimal impact of liquid lipid on zeta potential.
	Surfactant concentration	Stabilizers transfer charge, which is crucial for zeta potential.

Critical Process Parameters

The next group of potential factors influencing CQA variability is Critical Process Parameters, which have a special relationship with the manufacturing process of nano-formulations. Critical Process Parameters (CPPs) include key parameters that need to be kept within specified bounds in order to assure improved product quality. The QTPP and CQAs are directly impacted by these process parameters. The phase addition sequence, batch temperature, quantity, sonication speed and amplitude, stirring period and speed, homogenizing rate, and homogenizing period are a few examples of CPPs for nanocarriers [8]. The preliminary risk evaluation of the manufacturing process is presented in Table 5.

**Table 5:** Preliminary risk evaluation of the manufacturing process

	Process steps					
	Size reduction			Temperature	Stirring speed at the end of the process	Reference
Critical Quality Attributes of a drug product	Velocity of homogenization	Sonication intensity of the probe	Size diminution duration			
Size	Increased	Increased	Increased	Moderate	Moderate	[8]
Encapsulation efficiency	Increased	Increased	Moderate	Moderate	Decreased	[8]
Release of drug	Decreased	Decreased	Decreased	Increased	Decreased	[8]
Zeta potential	Decreased	Decreased	Decreased	Decreased	Increased	[8]

The justifications for the initial risk assessment of the manufacturing process are listed in Table 6.

**Table 6:** Justification for the initial risk assessment of the manufacturing process

Critical process attributes	Critical Quality Attributes of a drug product	Reasoning	Reference
Velocity of homogenization/sonication intensity of the probe	Size	A reduction in size significantly affects the dimensions of nanocarriers. Sonication amplitude and homogenization speed decrease their size.	[8]
	Encapsulation efficiency	For hydrophilic drugs, size reduction might lead to the drug leaking into an external phase.	
	Drug release	The process of size reduction does not impact drug release.	
	Zeta Potential	Size reduction does not affect the zeta potential.	
Size reduction time	Size	The duration of mixing and sonic treatment significantly influences the size.	[8]
	Encapsulation efficiency	The time required for a decrease in size has little effect on encapsulation efficacy.	
	Drug release	The process of size reduction does not influence drug release.	
	Zeta potential	Size reduction does not affect the zeta potential.	
Temperature	Size	Temperature affects size in a moderate way. Low temperature in lipid nanocarriers can cause big particles to develop and agglomerate.	[8]
	Encapsulation efficiency	The impact on entrapment efficiency is moderate. Low temperatures can prevent the entire lipid from melting, which could result in a decrease in entrapment efficiency.	
	Drug release	The impact of temperature on the release of drugs is moderate. When organic solvents evaporate at a high temperature, porous particles with rapid drug release are produced.	
	Zeta potential	Zeta potential is not much affected by temperature.	
Rate of agitating at the last step of the procedure	Size	Particle agglomeration results from low stirring speed, whereas small particle size creation is facilitated by high stirring speed.	[8]
	Encapsulation efficiency	Stirring speed barely affects the efficiency of entrapment.	
	Drug release	The impact of stirring speed on drug release is low.	
	Zeta potential	The impact of stirring speed on zeta potential is low.	

Safety and potential toxicity issues related to nanocarriers

Nanoparticles

The safety and good tolerance of nanocarriers are crucial considerations for advancing their use in treating eye conditions. Researchers evaluate the ocular toxicity of nanocarriers applied topically using the Draize test and histological studies. Although nanoparticles hold great potential in medicine, their extensive toxicity, highlighted in numerous studies, has constrained their usage. Therefore, rigorous monitoring is now essential to guarantee their safety. Two varieties of polycarboxylic acid nanoparticles loaded with brimonidine were assessed by De et al. [11] for their ability to lower intraocular pressure in glaucoma patients. They used human corneal epithelial cells to demonstrate the biocompatibility and non-toxicity of polyacrylic acid nanoparticles in vitro. In contrast, polyitanconic acid nanoparticles were observed to be harmful to the cells [11]. 

Meanwhile, Vega et al. [12] developed poly(lactic-*co*-glycolic acid) (PLGA) nanoparticles containing flurbiprofen. When administered topically to rabbits in vivo, these nanoparticles showed enhanced anti-inflammatory effects without causing irritation or damage to the adjacent ocular tissues [12]. Prow et al. [13] investigated the safety and toxicity of chitosan, amphiphilic polyphosphoester, and magnetic nanoparticles. They discovered that chitosan administered intravitreally causes ocular irritation. Nanoparticles present an added challenge due to their toxicity and size dynamics. Researchers observed toxicity in gold nanoparticles with a diameter of 1.4 nm, whereas those at a 15 nm diameter showed no toxicity. Additionally, studies indicate that nanoparticles can exhibit tissue toxicity depending on their shapes and morphology, as evidenced in carbon nanotubes [13]. 

Liposomes

Lajavardi et al. [14] found that PEGylated liposomes, which have a polyethylene glycol-coated surface, do not produce ocular inflammation in rats 24 hours after intravitreal injection, even at elevated levels (up to 116 mM lipid, or 1.16 μmol per eye). In addition, no damage was observed in the ocular region 14 days after 50 mM of this formulation was injected, according to a slit-lamp biomicroscopy investigation. As a result, a short- to mid-term intravitreal injection of these liposomes seems safe for the eye [14].

The local toxicity of medications delivered intravitreally by liposomes has been demonstrated to diminish in a number of animal model studies. Certain medications, such as those with anti-infective, anti-inflammatory, immune-suppressive or anti-metabolite characteristics, are less harmful in liposomal formulations than in their free versions. The decreased exposure of tissues to the drug molecules in their free state is responsible for this decrease in ocular toxicity [15].

Liposome-Nanoparticle Complex

Diebold et al. [16] synthesized combinations of liposomes and chitosan nanoparticles. These nanosystems were designed as eye drops capable of penetrating ocular mucosal barriers, combining the beneficial properties of both types of carriers. The scientists used a spontaneously immortalized epithelial cell line from a normal human conjunctiva ionically ordered bilayer assembly/N-hydroxysuccinimide carbonyl linker (IOBA-NHC) to evaluate the toxicity of the nanosystems. They measured cytotoxicity by the formation of a yellow color resulting from the cleavage of (2,3-bis(2-methoxy-4-nitro-5-sulfophenyl)-2H-tetrazolium-5-carboxanilide) (XTT) by mitochondrial enzymes. Compared to chitosan nanoparticles alone, the liposome-chitosan nanoparticle complexes showed higher cell viability. An in vivo acute tolerance test showed that the nanosystems did not exhibit any toxicity in either treated or sham-controlled eyes. The clinical microscopic sign score indicated the absence of any eye surface irritation [16].

Nanosuspension

A study comparing the efficacy and safety of flurbiprofen nanosuspension with over-the-counter eye drops was carried out by Boddeda et al. [17]. The flurbiprofen-loaded nanosuspension proved to be non-irritating and non-toxic based on the findings of the Draize evaluation and histological analysis [17].

Nanoemulsion

The safety of several cyclosporine-containing nanosuspensions, created to treat the ocular irritation brought on by Restasis®, was assessed using the Draize and Schirmer examinations. In the Draize assessment, the conjunctiva of both the commercial products and the nanosuspensions slightly reddened due to very little ocular discomfort. But in the Schirmer examination, the nanosuspension outperformed the commercial formulation, with no discernible differences in tear flow rates [18].

Niosomes

The MTT (3-(4,5-dimethylthiazol-2-yl)-2,5-diphenyltetrazolium bromide) test was employed to assess the potential toxicity of both the free drug and drug-loaded niosomal dispersions on a human corneal epithelial model. Hamad Alyami et al. [19] developed these niosomal formulations and discovered that niosomal formulations made with Span 60 reduced the cell toxicity produced by 2 mg/mL of the free drug. This was observed in both formulations with the surfactant: cholesterol ratios of 1:1 and 2:1. Despite having the largest particle sizes among the studied formulations, the Span 60 niosomes did not cause toxicity, confirming that niosomes smaller than 10 μm do not irritate the eyes. Despite having a sufficiently high entrapment efficiency, niosomal formulations made with Span 40 did not show significant differences in toxicity compared to the free drug. This suggests that the toxicity of the surfactant may contribute to the overall toxicity of the cell, even when the free drug is encapsulated [19].

**Figure 4:**
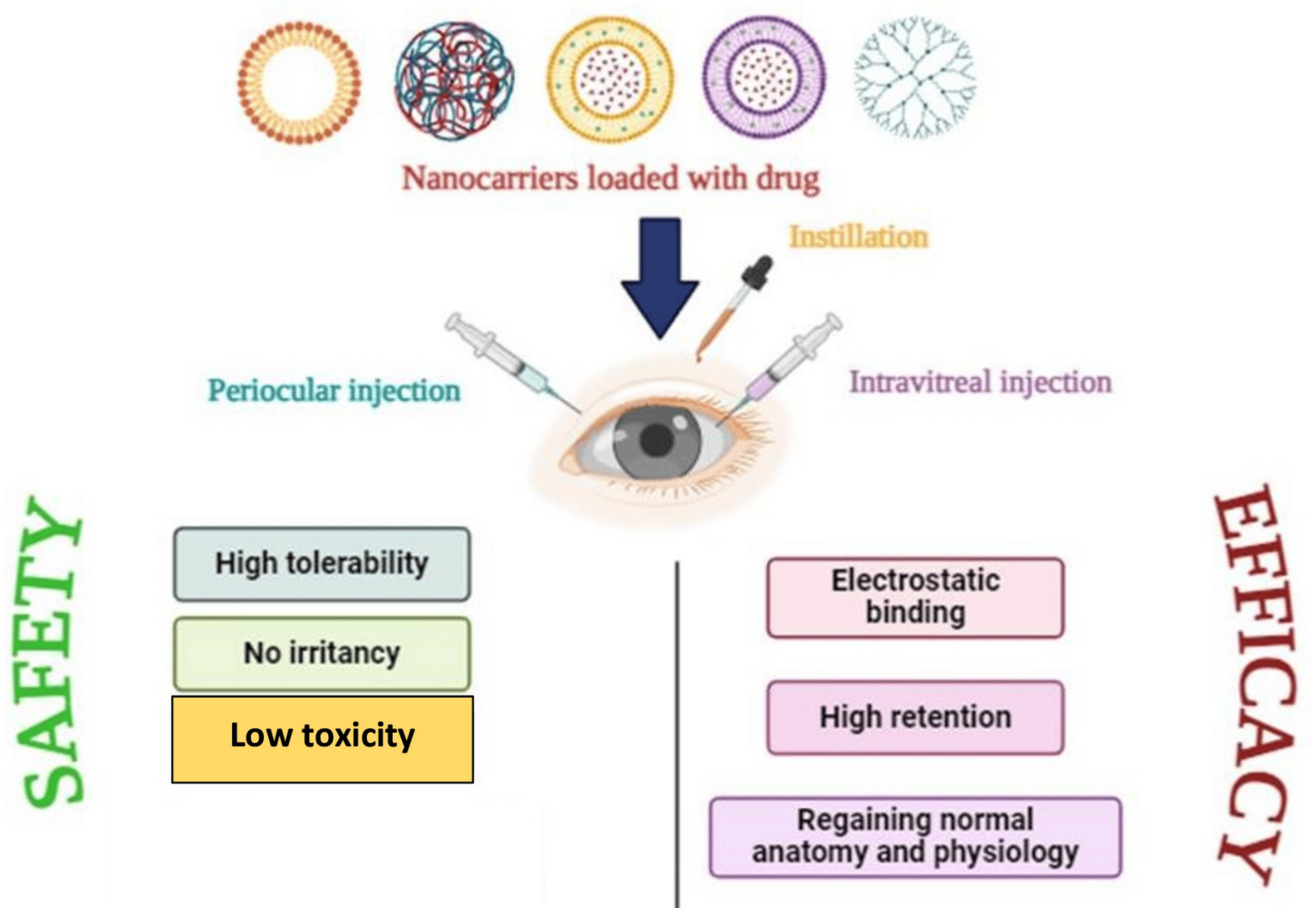
Safety and efficacy of nanocarriers used in ocular drug delivery system This image was created by Dr. Praiseth Bellston Rajan.

Due to the sensitivity of the eye to potential toxicity from nanocarriers and their component parts, it is crucial to confirm the safe use of ocular nanocarriers prior to when they are administered to patients [20]. The safety and efficacy of nanocarriers used in ocular drug delivery systems are presented in Figure 4. 

The marketed preparations of nanocarriers used in ophthalmology are listed in Table 7.

**Table 7:** Marketed preparations of nanocarriers used in ophthalmology. PMAM, Polyamidoamine.

Nanocarriers	Size	Description	Marketed preparations
Solid lipid nanoparticles	10 to 500 nm	They are non-toxic carriers and maintain long-term stability and prevent lipophilic drug decomposition. They easily modify the surface for targeted delivery of drugs.	Natacyn
Polymeric nanoparticles	<400 nm	Target-specific delivery of drugs. They increase treatment efficacy by avoiding non-specific distribution and reaching the correct ocular tissues. Drug degradation can be prevented. They enhance the absorption of drugs by boosting intracellular penetration.	Timoptic
Liposomes	0.08 to 10.00 µm	Water-soluble as well as fat-soluble drugs can be encapsulated. They are non-toxic and biocompatible. Corneal permeability can be improved.	Lipoquin
Niosomes	10 to 1000 nm	Drug delivery to ocular tissues with targeted distribution and controlled release, hence improving the drug's bioavailability. They are less toxic, biodegradable, biocompatible, and mucoadhesive.	Cyclopentol
Nanosuspensions	10 to 1000 nm	They enhance the bioavailability of ophthalmic medicines by improving their ability to dissolve. They also increase the duration of drug release and prolong the residency time in the cul-de-sac because it can make drugs that are poorly soluble in water more soluble in lacrimal fluid.	Rimoflo T Eye Drops, ILEVRO (Nepafenac ophthalmic suspension)
Nanostructured lipid carriers	50 to 1000 nm	They are biocompatible and stable and prevent the release of drugs when they are stored. They increase the drug's bioavailability to the ocular tissue.	Tetrandrine
Nanoemulsion	100 nm	They are clear and thermodynamically stable. They prolong the drug's release and increase its solubility, which lower the frequency of the dose. Corneal permeability is increased.	Cationorm ophthalmic emulsion, Durezol (difluprednate ophthalmic suspension)
Dendrimers	5 to 20 nm	Delivering lipophilic and hydrophilic drugs is feasible. They increases the solubility of the drugs and demonstrate significant drug loading and sustained release.	PAMAM (G1.5‑4.0), PAMAM core micelle

Drug delivery methods based on nanotechnology remain to be tested in clinical settings for ocular administration and they are provided in Table 8.

**Table 8:** Drug delivery methods based on nanotechnology remain to be tested in clinical settings for ocular administration. FDA, Food and Drug Administration; OCS, oral corticosteroids; AXR, Azelastine and Ketorolac; OTX, ophthalmic therapeutics extended.

Nanocarriers	Drug	Disease	Status	Clinical trials.gov Identifier (FDA)
Nanoparticles	Paclitaxel	Intraocular melanoma	II	NCT00738361
Nanoparticles	OCS‑01‑ dexamethasone cyclodextrin	Inflammation, corneal pain, postoperative	II	NCT04130802
Nanoparticles	Urea	Cataract	Randomized single-blind phase II trial	NCT03001466
Liposome	Latanoprost	Glaucoma	II	NCT02466399
Liposome	Vincristine	Aggressive tumor of the eye with metastases	II	NCT00506142
Nanoemulsion	Brimonidine tartrate	Dry eye disease	III	NCT03785340
Nanoemulsion	Cyclosporine	Dry eye disease	III	NCT04384991
Nanosuspension	Nepafenac	Diabetic macular edema	II	NCT01331005

Patents for ocular delivery methods for drugs based on nanotechnology are listed in Table 9.

**Table 9:** Patents for ocular delivery methods for drugs based on nanotechnology

S.No.	Patent number	Title of the patent	Disease focused	Year
1.	US10751337B2	Treatment strategies for dry eye disease and other eye conditions employing preservation-free ocular compositions	Various eye problems, including dry eye disease	2020
2.	CN110664757A	Application and preparation techniques for nanocrystalline eye drops	Birth-related macular degeneration	2020
3.	KR20200000395A	A cyclosporine-containing eye composition and a method of preparation	A tingling feeling in the eye, conjunctivitis, and dry eye disease	2020
4.	WO2018233878A1	A clobetasol nanoemulsion composed of oil and water	Disorders or diseases that cause inflammation	2019
5.	CN109906075A	Liposome corticosteroid for local administration to an inflammatory lesion or area	Eye lesion with inflammation	2019
6.	JP2019108372A	Nitro phenylboronic acid mixture stabilizes nanoparticles	To efficiently administer drugs to particular eye targets	2019
7.	US20190070302A1	The polymer and drug combine to generate nanoparticles, implants, and microparticles.	Uveitis and wet age-related macular degeneration (AMD)	2019
8.	US9931306B2	Conjunctival cul-de-sac using a nanostructured biocompatible wafer	The ocular surface disease glaucoma	2018
9.	US9956195B2	Liposomes containing a minimum of one lipid bilayer, such as phosphatidylcholine and prostaglandin F2α	Dry eye condition	2018
10.	US 10,010,516 t	Technique for controlling the vitality of retinal endothelial cells	Pathological circumstances affecting retinal endothelial cells	2018
11.	AU2013256092B2	Pharmaceutical nanoparticles with enhanced mucosal transport	Assist in the delivery of less soluble drugs directly into the eye	2017
12.	WO2017152129A2	Therapy for retinal illnesses and/or glaucoma	Disorders of the retina and glaucoma	2017
13.	US 9,320,801 B2	Non irritative nanoemulsion ophthalmic formulation containing cyclosporine	Dry eye condition	2016
14.	CN108066315A	The preparation process for ophthalmic puerarin and scutellarin lipid nanoparticles	Cataract	2016
15.	CN105726484B	Tetrandrine ophthalmic formulation and preparation technique using seed crystal nanoparticles	Cataract	2016

Future perspectives

The recent surge in ocular nanotherapies is largely due to increased collaborations between material researchers and healthcare workers who identify specific clinical needs addressable by material science tools, facilitating rapid translation to clinical applications. Despite advancements in nanotechnology-based strategies for ocular topical therapy, there remains a critical need to develop novel formulations with reduced dosage and administration frequency, enhanced drug release, and action at the target area, and fewer adverse events. The primary focus has shifted toward nanoparticles for topical application and subconjunctival implants, prompting diversification for broader benefits. Future research should prioritize developing safer, more effective nano-formulations capable of delivering small molecules and biologics (such as genes and peptides) to the anterior eye segment. These formulations should also ensure reduced toxicity, high stability, and improved pharmacokinetic and pharmacodynamic properties of the delivered drugs.

Liposomes have been extensively studied for ocular drug delivery; however, their high cost and rapid clearance from circulation by cells present challenges. Polymeric nanoparticles, on the other hand, offer promising alternatives as they can be easily functionalized with various drugs, are biodegradable, and pose minimal harm. Among them, polymers based on poly(lactic-*co*-glycolic acid) (PLGA) have been effectively utilized in ocular drug delivery systems. Future efforts in nanoparticle development aim to enhance target specificity, prolong the duration of action, and minimize adverse effects.

Nanotechnology represents a significant advancement over traditional ocular drug delivery methods, offering the potential for increased therapeutic efficacy and innovative functionalities. The market for pharmaceuticals used to treat eye illnesses is expected to increase at an average annual rate of around 4.7% between 2018 and 2024, from USD 25.03 billion in 2017 to USD 34.52 billion by the end of 2024. This growth trajectory indicates substantial opportunities for novel therapeutic applications in the near future within the ocular product sector.

## Conclusions

The most effective and patient-friendly treatment for ocular diseases is local therapy, owing to the complicated anatomy and physiology of the eye. There is a lot of interest in the topic because targeted medication delivery using nanomedicine to both the front and back of the ocular region aims to address the drawbacks of traditional preparations. The treatment for eye illnesses has become easier due to the use of nanocarriers for controlled and site-specific medication distribution to ocular components. This approach could help patients with eye disorders live better lives, experience less discomfort, and require fewer intravitreal injections. It may also increase treatment efficiency and reduce treatment expenses. Promising techniques such as membrane extrusion, supercritical fluid technology, and microfluidizers offer scalability for manufacturing these advanced drug delivery systems. Several ophthalmic nanomedicines, including hydrogels, advanced Particle Replication In Nonwetting Templates, liposomes (PRINT), nanocrystals, and nanoemulsions, have already been commercialized or are in development, highlighting their potential to revolutionize ocular drug delivery.

Nanotechnology has garnered significant research focus in the United States, accounting for one-third of all publications and half of all patent filings in recent years. With a projected compound annual growth rate (CAGR) of 22%, the nanotechnology sector represents a promising area for investment. Numerous medication delivery devices are currently undergoing clinical trials, and the nanomedicine industry is still in its early stages of development. The Food and Drug Administration has already approved several intravitreal polymeric drug delivery implants for treating eye disorders. In conclusion, the advantages of novel drug delivery systems such as drug targeting, sustainability, and enhanced bioavailability are undeniable for ocular applications. Therefore, these innovative nanocarriers are expected to see increasing utilization in clinical practice in the future.
